# *Saccharomyces boulardii* Administration Changes Gut Microbiota and Attenuates D-Galactosamine-Induced Liver Injury

**DOI:** 10.1038/s41598-017-01271-9

**Published:** 2017-05-02

**Authors:** Lei Yu, Xue-ke Zhao, Ming-liang Cheng, Guo-zhen Yang, Bi Wang, Hua-juan Liu, Ya-xin Hu, Li-li Zhu, Shuai Zhang, Zi-wen Xiao, Yong-mei Liu, Bao-fang Zhang, Mao Mu

**Affiliations:** 1 Prenatal Diagnosis Center, Hospital Affiliated to Guizhou Medical University, NO. 4 Beijing Road, Guiyang, 550004 Guizhou China; 2 Department of Infectious Diseases, Hospital Affiliated to Guizhou Medical University, NO. 4 Beijing Road, Guiyang, 550004 Guizhou China; 3 Department of Eugenics and Genetics, Guiyang Maternal and Child Health-Care Hospital, Ruijin South Road 63, Guiyang, 550003 Guizhou China; 4 Department of Interventional Radiology, Cancer Hospital of Guizhou Medical University, NO. 1 West Beijing Road, Guiyang, 550004 Guizhou China

## Abstract

Growing evidence has shown that gut microbiome is a key factor involved in liver health. Therefore, gut microbiota modulation with probiotic bacteria, such as *Saccharomyces boulardii*, constitutes a promising therapy for hepatosis. In this study, we aimed to investigate the protective effects of *S. boulardii* on D-Galactosamine-induced liver injury in mice. Liver function test and histopathological analysis both suggested that the liver injury can be effectively attenuated by *S. boulardii* administration. In the meantime, *S. boulardii* induced dramatic changes in the gut microbial composition. At the phylum level, we found that *S. boulardii* significantly increased in the relative abundance of *Bacteroidetes*, and decreased the relative abundance of *Firmicutes* and *Proteobacteria*, which may explain the hepatic protective effects of *S. boulardii*. Taken together, our results demonstrated that *S. boulardii* administration could change the gut microbiota in mice and alleviate acute liver failure, indicating a potential protective and therapeutic role of *S. boulardii*.

## Introduction

Acute liver failure (ALF) is a rare and life-threatening disorder with extremely high short-term morbidity and mortality^1^. ALF can be caused by a variety of conditions, such as drug-induced liver injury (DILI)^2–4^, acute viral infections from hepatitis^5, 6^ and autoimmune diseases^7^. Regardless of the etiology, ALF can be associated with rapid deterioration and devastating complications^8^. Although advances in liver transplantation have improved survival of ALF cases in recent years, mortality remains significant. The limited availability of liver transplantation and the urgent need of waitlisted patients have led to great interest in the development of alternative therapies. Transplant-free survival in ALF has increased considerably with more and more cases recovered with supportive medical care alone^9^.

Microbes in the gastrointestinal tract, referred to as gut microbiota, have a collective genome with 150-fold more genes than the human genome^10^. Rapid advances of biotechnology have markedly improved our understanding of the role played by gut microbiota in health and disease^11–14^. Recent literatures suggest that qualitative changes in the gut microbiota, such as increased levels of harmful bacteria or reduced proportions of beneficial bacteria, are associated with pathogenesis and progression of liver disorders^15, 16^. Such studies lead to a general hypothesis that administration of health-promoting microbial strains may help treat liver diseases^17, 18^. Therefore, a number of beneficial bacteria are tested in animal models and exhibited therapeutic effects on alcoholic liver disease^19^, acute liver injury^20^, liver fibrosis^21^, and non-alcoholic fatty liver disease^22^.

*Saccharomyces boulardii* (*S. boulardii*) is a selected strain of nonpathogenic yeast, which is commercialized worldwide as a probiotic for humans^23^. A great number of clinical trials and pre-clinical studies demonstrate the efficacy and safety of *S. boulardii* for various disease indications^24, 25^, such as irritable bowel syndrome, Crohn’s disease, and diarrhea with different causes. Recently, further evidence suggests that *S. boulardii* can promote the liver function and ameliorate liver fibrosis^26^, hepatic steatosis^27^, and hepatic injury induced by infection^28^. However, the underlying mechanisms of such protection remain largely unclear.

Thus, this study has the following aims: (i) to examine the influence of *S. boulardii* on liver function and hepatocyte architecture in mouse model of D-Galactosamine (D-GalN) induced liver injury and (ii) to investigate the impact of *S. boulardii* administration on the taxonomic composition of the mouse gut microbiota by utilizing high-throughput sequencing technology.

## Materials and Methods

### Animals and tissue sampling

Experiments were performed on adult BALB/c mice. The mice were individually housed in plastic cages at room temperature (22 °C) and maintained on an artificial cycle of 12-h light and 12-h dark. Surgical preparations involved anesthetization with a xylazine/ketamine mixture. The mice were then sacrificed by cervical dislocation. Liver tissue was precisely dissected, immersed in liquid nitrogen, and stored at −80 °C until further analysis. All procedures were approved by the Ethics Committee of Hospital Affiliated to Guizhou Medical University and performed in accordance with the guidelines on animal ethics.

### Experimental design

D-Galactosamine (D-GalN) was purchased from Sigma Aldrich Corporation (St. Louis, MO, USA). *S. boulardii* was purchased from Biocodex (France). After ruling out baseline differences in blood and fecal samples, the experimental mice were randomly divided into three groups (n = 5 per group): (1) mice that served as vehicle control (CTRL group), (2) mice that were treated with D-GalN (D-GalN group), and (3) mice that were treated with D-GalN and probiotic *S. boulardii* (D-GalN + SB group). The D-GalN + SB group were gavaged with 1 ml of *S. boulardii* (1 × 10^9^ CFU/ml) for 4 weeks prior to exposure to D-GalN. And the CTRL group and D-GalN group received the same volume of saline solution. D-The GalN group and the D-GalN + SB group were then intraperitoneally (i.p.) injected with 200 mg/kg D-GalN, while the CTRL group were injected with saline solution. All the mice were sacrificed 24 h after D-GalN challenge. Serum samples, liver tissue specimens and gut microbiota were compared between groups.

### Serum aminotransferase activities

Fasting blood was collected from each mouse and centrifuged at 1,000 × g for 5 min at room temperature. Then, the serum sample was extracted and stored at −20 °C until further analysis. Serum alanine aminotransferase (ALT) and aspartate aminotransferase (AST) activities were quantified with the enzymatic kinetic method by using a semi-automatic biochemistry analyzer (HORRON RD171; HORRON XLH Medical Electronics) according to the manufacturer’s protocol.

### Histologic examination

Liver tissue specimens measuring approximately 0.2 cm × 0.2 cm were taken from the right lobe of liver of each mouse. All specimens were dehydrated through graded solutions of alcohol, fixed in pH 7.4 and 10% buffered neutral formalin, and embedded in molten paraffin wax. After hematoxylin and eosin staining, the morphologic evaluation was carried out with a light microscope (SP2, Leica).

### Taxonomic microbiota analysis

Metagenomic DNA was extracted from the ileal contents of mice by using the QIAamp Fast DNA Stool Mini Kit (Qiagen) following the manufacturer’s guidelines and previously published protocols^29, 30^. Real-time q-PCR was performed using TaqMan® Universal Master Mix (Life technologies) to examine the quality and the quantity of the 16 S rDNA. The variable region 4–6 (V4–V6) of the purified 16 S rDNA gene was amplified with PCR. Sequencing was performed utilizing paired-end Illumina MiSeq sequencing system and reagents according to the manufacturer’s instructions. Raw FASTQ files reflecting forward reads were initially filtered for quality and length (≥200 bp) using QIIME^31^. Passing sequences were trimmed of the forward primer, and evaluated for chimeras with UCHIME^32^. The RDP Classifier software was used to bin 16 S rRNA gene sequences into operational taxonomic units (OTUs), which were defined by clustering at 97% similarity.

### Statistical analysis

All analyses were performed on non-rarefied data by using R 3.2.5 with relevant packages. Community ordination analysis were performed using weighted UniFrac distances^33^ and principal coordinates analysis (PCoA), so as to visualize the difference in bacterial populations between groups. Biochemical experimental results are expressed as mean ± SEM. And values of AST and ALT activities were logarithmically transformed to approximate a normal distribution. Differences in bacterial relative abundance between groups were assessed at phylum and family levels. Independent two-tailed Student’s t-test was performed following previously published procedures^34, 35^. All p-values were adjusted for multiple using the Benjamini-Hochberg method. Adjusted p-values with false-discovery rate (FDR) below 0.05 were considered significant.

## Results

### Serum AST and ALT activities

**Figure 1:**
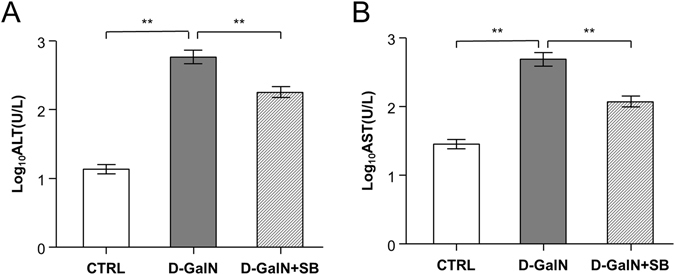
Effects of D-GalN treatment and *S. boulardii* supplementation on ALT (**A**) and AST (**B**) activities. Values are displayed as means ± SEMs. *P-value < 0.05 according to Student’s t-test.

Three groups of mice were studied, including the CTRL group (i.e., vehicle control), the D-GalN group with D-Galactosamine-induced liver injury, and the D-GalN + SB group with *S. boulardii* treatment before D-GalN challenge (see Materials and Methods). The plasma levels of ALT and AST were measured as an indicator of D-GalN-induced liver injury (Fig. 1). Compared with the CTRL group, there was a significant elevation in the levels of ALT and AST in the D-GalN group (P < 0.01 for ALT, P < 0.01 for AST), indicating massive abnormality in liver function. On the other hand, the levels of ALT and AST of the D-GalN + SB group were substantially lower than those of the D-GalN group (P < 0.01 for ALT, P < 0.01 for AST), suggesting marked attenuation in liver injury after *S. boulardii* administration.

### Histopathological analysis of liver sections

**Figure 2:**
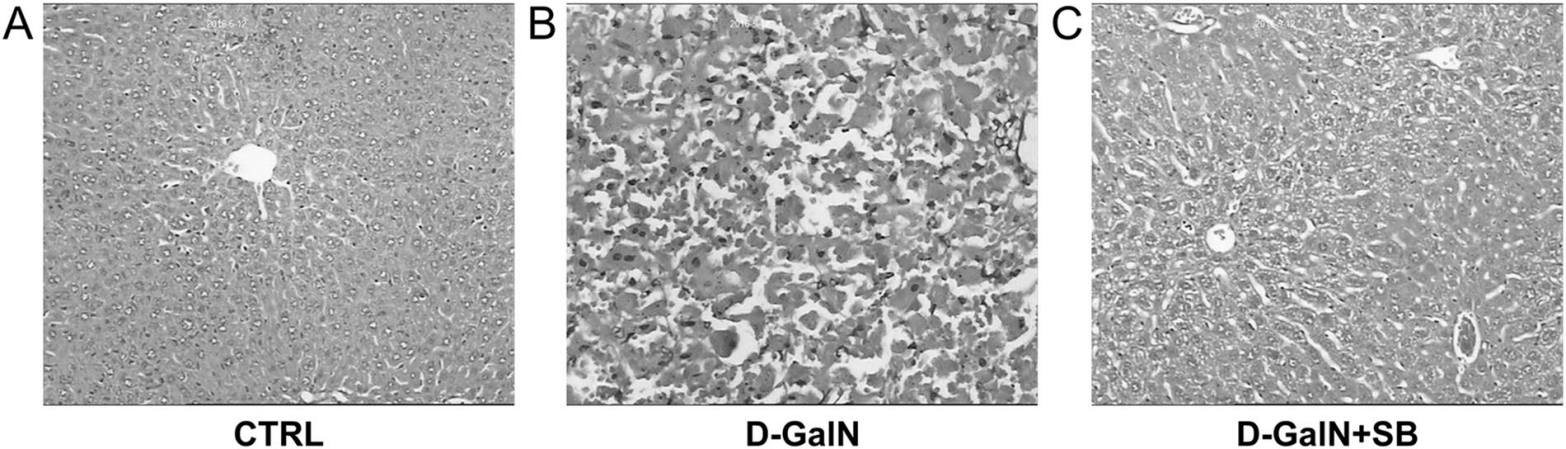
Histological analysis of liver sections. (**A**) Histology of the CTRL group with normal liver architecture. (**B**) The D-GalN group exhibited severe hemorrhage, inflammation, and necrosis of hepatocytes. (**C**) Hepatic injury was attenuated in the D-GalN + SB group.

Histology of the rat liver sections (see Materials and Methods) exhibited a normal lobular liver architecture and integrated cell structure in CTRL group (Fig. 2A). Challenge with D-GalN resulted in acute hepatic injury accompanied by prominent hemorrhage and inflammation, necrosis of hepatocytes, and serious dissolution of the hepatocyte architecture (Fig. 2B). Such liver alterations were apparently alleviated in the D-GalN + SB group (Fig. 2C). Such differences indicated that *S. boulardii* supplementation indeed mitigated D-GalN-induced liver injury.

### Gut microbiota profoundly affected by *S. boulardii*

**Figure 3:**
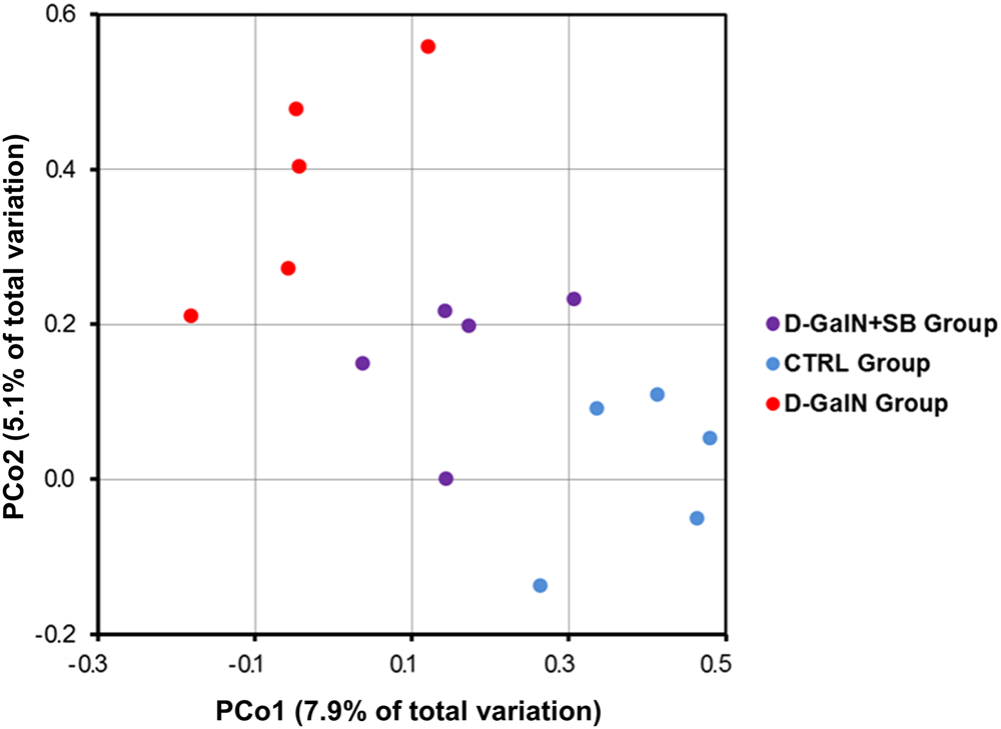
PCoA analysis for the gut bacterial community based on the weighted Unifrac distance between the CTRL group (blue dots), the D-GalN group (red dots) and the D-GalN + SB group (purple dots).

Liver function test and histopathological analysis both suggested that the liver injury induced by D-GalN can be effectively attenuated by *S. boulardii* administration. In order to further study the underlying mechanisms for the protective effects of *S. boulardii* on liver, the metagenomic DNA was quantified and mapped into operational taxonomic units (OTUs; see Materials and Methods). The ileal contents was selected for DNA extraction, since it was reported that ileal samples had much lower inter-mouse variation than those from other tissue samples^36^. Principal coordinates analysis (PCoA) was performed to visualize the difference between individual treatment groups (Fig. 3). In general, D-GalN treated mice showed a clear separation from the vehicle controls. However, the mice under *S. boulardii* supplementation were less affected and displayed a relatively restored gut microbial composition after D-GalN challenge.

**Figure 4:**
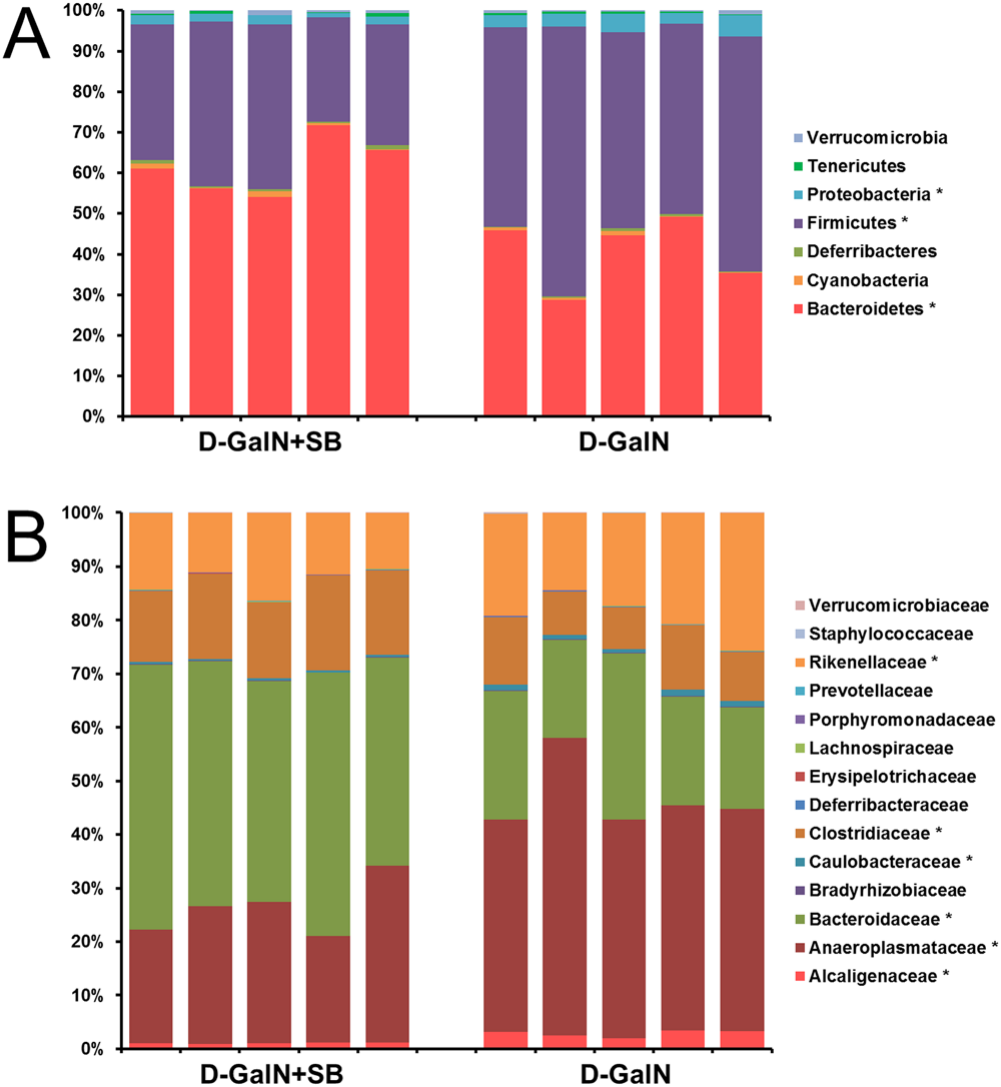
Variation in bacterial community composition in the ileum at phylum (**A**) and family (**B**) levels. Undetected taxa are not displayed in the chart. *P-value < 0.05 according to Student’s t-test.

We further compared the D-GalN group and the D-GalN + SB group, so as to scrutinize how *S. boulardii* supplementation profoundly affected the abundance of different phyla and families. At the phylum level, we found that *S. boulardii* was associated with a significant increase in the relative abundance of *Bacteroidetes* (61.7% vs 40.8%) and a significant decrease in *Firmicutes* (33.9% vs 53.7%) and *Proteobacteria* (1.9% vs 3.7%) compared to the D-GalN group (Fig. 4A; see also Table S1). These results suggested that *S. boulardii* changed the gut microbial community by altering the proportion of three major phyla. At the family level, we also observed several important modifications of the gut microbial composition. Among the major families identified, *Bacteroidaceae* and *Clostridiaceae* were significantly increased following *S. boulardii* treatment. Conversely, *Alcaligenaceae*, *Anaeroplasmataceae*, *Caulobacteraceae* and *Rikenellaceae* were decreased (Fig. 4B; see also Table S2).

## Discussion

Recently, much attention is paid to the research on probiotics as an adjuvant for the prevention or treatment of gastrointestinal diseases. There are certain advantages of probiotic therapy. For instance, probiotics have few side effects and drug resistance. Also, compared with many other therapies, the cost of probiotics is relatively low. A series of previous studies demonstrate that the gut microbiota is closely associated with the development of hepatic steatosis and inflammation^37, 38^. *S. boulardii*, as a tropical species of yeast isolated from fruits, is widely used to introduce beneficial active cultures into the intestine and confer protection against pathogenic microorganisms. However, many details about the impact of *S. boulardii* on hepatic pathology and gut microbiota remain largely unknown. Thus, we used animal models and high-throughput sequencing method to investigate (i) the impact of *S. boulardii* on the integrity of liver tissue and hepatic function and (ii) the effect of *S. boulardii* on gut microbiota composition.

The assessment of histopathological changes and aminotransferase activities demonstrated that D-GalN- induced hepatic injury could be alleviated by *S. boulardii* intervention. In the liver, ALT is normally enriched in the cytoplasm of hepatocytes, and AST is located in both the cytoplasm and mitochondria. When the hepatic architecture is damaged by D-GalN, ALT and AST are released into the serum, leading to acutely increased activities. In the present study, the fact that *S. boulardii* significantly reduced the release of hepatic ALT and AST indicated an attenuated liver injury, which was also directly corroborated by histopathological observation. A similar protective role of *S. boulardii* against chronic hepatic steatosis was also observed in diabetic mouse model^27^. Here we further found that *S. boulardii* can evidently alleviate acute liver injury, which merits in-depth pathological and pharmacological study.

This study also demonstrated that *S. boulardii* intervention in D-GalN-treated mice profoundly changed the gut microbial composition. After *S. boulardii* administration, mice exhibited alleviated necrosis of hepatocytes, hemorrhage and inflammatory infiltration, thereby suggesting that *S. boulardii* may act as a beneficial probiotic in the context of acute liver injury. We identified alterations in the relative proportion of *Bacteroidetes*, *Firmicutes* and *Proteobacteria* phyla in ileal bacterial communities after *S. boulardii* intervention. Decreased abundance of *Bacteroidetes* and increased abundance of *Firmicutes* or *Proteobacteria* have been reported to be associated with a variety of hepatosis, including liver cirrhosis^39^, nonalcoholic steatohepatitis^40^ and nonalcoholic fatty liver disease^41^. The fact that *S. boulardii* could reverse the above bacterial imbalances may explain the hepatic protective effects of *S. boulardii*. At the family level, we also identified a number of bacterial families affected by *S. boulardii* treatment. Importantly, many of them remain poorly understood and could be novel bacteria to study in the context of liver injury, since we should not rule out the possibility that changes in specific bacterial families are involved in the beneficial effects of *S. boulardii* on liver function.

Although *S. boulardii* supplementation was observed to alter gut microbial composition and alleviate D-GalN-induced acute liver injury, we still lack insights into the interaction between gut bacterial community and liver function. Emerging evidence has suggested that intestinal bacteria play a key role in maintaining the health of gut-liver axis^42, 43^. Thus, gut microbiota modulated by *S. boulardii* intervention may represent a new way to treat acute liver injury. Microarray has been successfully applied to establish a global transcriptomic profile for injury and regeneration of D-GalN-administered mouse livers^44^. Therefore, we plan to investigate how *S. boulardii* alters gene expression in liver, so as to unveil the molecular mechanisms underlying its liver-protecting effect. For instance, it has been widely recognized that inflammatory microenvironment can profoundly influence the pathogenesis of liver fibrosis^45^. We will investigate relevant genes, especially pro-inflammatory cytokines^46^, to better understand how *S. boulardii* ameliorates inflammation in liver. In addition, it must be recognized that animal models may not fully represent the hepatic pathology in humans, as suggested by extensive studies showing positive results in rodents but hardly translated into humans^47, 48^. Even though *S. boulardii* may be beneficial in attenuating D-GalN-induced liver injury, further clinical research is required to validate such protective effect.

In summary, this study provided an in-depth analysis on the gut microbiota modulations that occurred after *S. boulardii* supplementation. Our results demonstrated that *S. boulardii* administration could change the gut microbiota in mice and alleviate D-GalN-induced acute liver injury, indicating a potential therapeutic role of *S. boulardii*. Further transcriptomic and clinical research is required to better understand the underlying mechanisms of the hepatic protective effects of *S. boulardii*.

## Electronic supplementary material

Table S1
