# Supplementary material for: Saccharomyces boulardii Administration Changes Gut Microbiota and Attenuates D-Galactosamine-Induced Liver Injury
Source: Sci Rep. 2017 May 2;7:1359. doi: 10.1038/s41598-017-01271-9 (PMC5430957; doi:10.1038/s41598-017-01271-9)
Supplement: Supplementary file 1 — Table S1 [file 41598_2017_1271_MOESM1_ESM.doc]

***Saccharomyces boulardii* Administration Changes Gut Microbiota and Attenuates D-Galactosamine-Induced Liver Injury**

Lei Yu^1, #^, Xu-ke Zhao^2, #^, Ming-liang Cheng^2,*^, Guo-zhen Yang^1,*^, Bi Wang^1, 3^, Hua-juan Liu^2^, Ya-xin Hu^2^, Li-li Zhu^2^, Shuai Zhang^4^, Zi-wen Xiao^1^, Yong-mei Liu^2^, Bao-fang Zhang^2^, Mao Mu^2^

^1^ Prenatal Diagnosis Center, Hospital Affiliated to Guizhou Medical University, NO. 4 Beijing Road, Guiyang 550004, Guizhou, China.

^2^ Department of Infectious Diseases, Hospital Affiliated to Guizhou Medical University, NO. 4 Beijing Road, Guiyang 550004, Guizhou, China.

^3^ Department of Eugenics and Genetics, Guiyang Maternal and Child Health-Care Hospital, Ruijin South Road 63, Guiyang 550003, Guizhou, China.

^4^ Department of Interventional Radiology, Cancer Hospital of Guizhou Medical University, NO. 1 West Beijing Road, Guiyang 550004, Guizhou, China.

^#^ These authors contributed equally to this work.

^*^ Correspondence and requests for materials should be addressed to Ming-liang Cheng (email: gmcmingliang_cheng@163.com) or Guo-zhen Yang (email: gmcguozhen_yang@163.com).

**Table S1.** Relative proportion of different phyla in ileal contents.

|  |  | **D-GalN+SB Group** | | | | | |  | **D-GalN Group** | | | | | |  | **Adjusted P-value** |
| --- | --- | --- | --- | --- | --- | --- | --- | --- | --- | --- | --- | --- | --- | --- | --- | --- |
|  |  | **Sample 1** | **Sample 2** | **Sample 3** | **Sample 4** | **Sample 5** | **Mean** |  | **Sample 1** | **Sample 2** | **Sample 3** | **Sample 4** | **Sample 5** | **Mean** |  |
| ***Bacteroidetes*** | | 61.% | 56.2% | 54.1% | 71.8% | 65.6% | 61.7% |  | 45.9% | 28.7% | 44.7% | 49.2% | 35.4% | 40.8% |  | 0.012 |
| ***Cyanobacteria*** | | 1.2% | 0.1% | 1.4% | 0.5% | 0.2% | 0.7% |  | 0.7% | 0.6% | 0.9% | 0.1% | 0.2% | 0.5% |  | 0.70 |
| ***Deferribacteres*** | | 1.0% | 0.4% | 0.5% | 0.4% | 1.0% | 0.7% |  | 0.1% | 0.3% | 0.8% | 0.4% | 0.2% | 0.4% |  | 0.22 |
| ***Firmicutes*** | | 33.4% | 40.5% | 40.5% | 25.7% | 29.7% | 33.9% |  | 49.1% | 66.5% | 48.3% | 47.0% | 57.8% | 53.7% |  | 0.012 |
| ***Proteobacteria*** | | 2.2% | 2.0% | 2.3% | 1.0% | 1.9% | 1.9% |  | 3.0% | 3.2% | 4.6% | 2.5% | 5.2% | 3.7% |  | 0.048 |
| ***Tenericutes*** | | 0.4% | 0.7% | 0.1% | 0.2% | 1.0% | 0.5% |  | 0.4% | 0.4% | 0.4% | 0.4% | 0.3% | 0.4% |  | 0.70 |
| ***Verrucomicrobia*** | | 0.8% | 0.1% | 1.1% | 0.4% | 0.6% | 0.6% |  | 0.7% | 0.4% | 0.3% | 0.3% | 0.9% | 0.5% |  | 0.70 |

**Table S2.** Relative proportion of different families in ileal contents.

|  |  | **D-GalN+SB Group** | | | | | |  | **D-GalN Group** | | | | | |  | **Adjusted P-value** |
| --- | --- | --- | --- | --- | --- | --- | --- | --- | --- | --- | --- | --- | --- | --- | --- | --- |
|  |  | **Sample 1** | **Sample 2** | **Sample 3** | **Sample 4** | **Sample 5** | **Mean** |  | **Sample 1** | **Sample 2** | **Sample 3** | **Sample 4** | **Sample 5** | **Mean** |  |
| ***Alcaligenaceae*** | | 1.1% | 0.9% | 1.1% | 1.2% | 1.2% | 1.1% |  | 3.1% | 2.5% | 1.9% | 3.5% | 3.3% | 2.9% |  | 0.0092 |
| ***Anaeroplasmataceae*** | | 21.2% | 25.8% | 26.3% | 19.8% | 32.9% | 25.2% |  | 39.7% | 55.5% | 40.9% | 42.0% | 41.6% | 43.9% |  | 0.0084 |
| ***Bacteroidaceae*** | | 49.4% | 45.7% | 41.3% | 49.1% | 38.9% | 44.9% |  | 24.0% | 18.3% | 31.1% | 20.3% | 18.9% | 22.5% |  | 0.0016 |
| ***Bradyrhizobiaceae*** | | 0.1% | <0.1% | 0.1% | <0.1% | <0.1% | <0.1% |  | 0.1% | 0.1% | 0.1% | 0.1% | 0.1% | 0.1% |  | 0.098 |
| ***Caulobacteraceae*** | | 0.4% | 0.3% | 0.4% | 0.4% | 0.5% | 0.4% |  | 1.1% | 0.9% | 0.7% | 1.2% | 1.2% | 1.0% |  | 0.0092 |
| ***Clostridiaceae*** | | 13.3% | 15.9% | 14.3% | 17.7% | 15.8% | 15.4% |  | 12.6% | 8.0% | 7.9% | 12.1% | 9.1% | 10.0% |  | 0.0092 |
| ***Deferribacteraceae*** | | <0.1% | <0.1% | <0.1% | <0.1% | 0.1% | <0.1% |  | 0.1% | 0.1% | 0.1% | <0.1% | <0.1% | 0.1% |  | 0.43 |
| ***Erysipelotrichaceae*** | | 0.1% | 0.1% | <0.1% | <0.1% | 0.1% | 0.1% |  | <0.1% | <0.1% | <0.1% | <0.1% | 0.1% | <0.1% |  | 0.42 |
| ***Lachnospiraceae*** | | 0.1% | 0.1% | <0.1% | <0.1% | 0.1% | <0.1% |  | <0.1% | 0.1% | 0.1% | <0.1% | 0.1% | <0.1% |  | 0.91 |
| ***Porphyromonadaceae*** | | <0.1% | 0.1% | <0.1% | 0.1% | <0.1% | <0.1% |  | 0.1% | <0.1% | <0.1% | <0.1% | 0.1% | <0.1% |  | 0.75 |
| ***Prevotellaceae*** | | 0.1% | <0.1% | 0.1% | <0.1% | 0.1% | <0.1% |  | 0.1% | 0.1% | <0.1% | <0.1% | <0.1% | <0.1% |  | 0.87 |
| ***Rikenellaceae*** | | 14.1% | 11.0% | 16.3% | 11.4% | 10.3% | 12.6% |  | 19.0% | 14.3% | 17.2% | 20.7% | 25.5% | 19.3% |  | 0.046 |
| ***Staphylococcaceae*** | | 0.1% | <0.1% | <0.1% | <0.1% | 0.1% | 0.1% |  | 0.1% | <0.1% | 0.1% | <0.1% | 0.1% | 0.1% |  | 0.57 |
| ***Verrucomicrobiaceae*** | | <0.1% | 0.1% | 0.1% | <0.1% | 0.1% | <0.1% |  | 0.1% | <0.1% | <0.1% | 0.1% | <0.1% | <0.1% |  | 0.87 |
